# Graphitic Porous Carbon Derived from Waste Coffee Sludge for Energy Storage

**DOI:** 10.3390/ma13183972

**Published:** 2020-09-08

**Authors:** Hyeyoung Jung, Jihyeon Kang, Inho Nam, Sunyoung Bae

**Affiliations:** 1 Department of Chemistry, Seoul Women’s University, Seoul 01797, Korea; jyoung3686@naver.com; 2 School of Chemical Engineering and Materials Science, Department of Intelligent Energy and Industry, Institute of Energy Converting Soft Materials, Chung-Ang University, Seoul 06974, Korea; kar04114@cau.ac.kr

**Keywords:** recycling, coffee sludge, hydrothermal carbonization, hydrochar, electrical double-layer capacitor (EDLC)

## Abstract

Coffee is one of the largest agricultural products; however, the majority of the produced coffee is discarded as waste sludge by beverage manufacturers. Herein, we report the use of graphitic porous carbon materials that have been derived from waste coffee sludge for developing an energy storage electrode based on a hydrothermal recycling procedure. Waste coffee sludge is used as a carbonaceous precursor for energy storage due to its greater abundance, lower cost, and easier availability as compared to other carbon resources. The intrinsic fibrous structure of coffee sludge is based on cellulose and demonstrates enhanced ionic and electronic conductivities. The material is primarily composed of cellulose-based materials along with several heteroatoms; therefore, the waste sludge can be easily converted to functionalized carbon. The production of unique graphitic porous carbon by hydrothermal carbonization of coffee sludge is particularly attractive since it addresses waste handling issues, offers a cheaper recycling method, and reduces the requirement for landfills. Our investigations revealed that the graphitic porous carbon electrodes derived from coffee sludge provide a specific capacitance of 140 F g^−1^, with 97% retention of the charge storage capacity after 1500 cycles at current density of 0.3 A g^−1^.

## 1. Introduction

Coffee is one of the common agricultural products that are generally used for beverages. The global production of coffee is approximately 120 billion bags per year (60 kg per bag) from the International Coffee Organization [1], which corresponds to an annual production of approximately eight million metric tons of coffee; therefore, coffee can be regarded as an important agricultural commodity [2]. However, most of the produced coffee is discarded as waste sludge by beverage manufacturers. In 2016, the average annual coffee consumption in South Korea was 377 cups per adult, and the corresponding increase in the annual average since 2012 has been 7%. To prepare a cup of coffee, only 0.2% of the coffee beans are used and the remaining 99.8% is thrown away as waste coffee sludge; further, it is estimated that over 124,000 tons of waste are generated [3] annually.

The average oil content in the waste coffee sludge has been determined to be 15%, which can be converted to a similar amount of biodiesel via transesterification methods [1]. Excluding the oil components, coffee sludge is primarily composed of cellulose-based materials with many heteroatoms (e.g., nitrogen, oxygen, and sulfur), which are considered good precursors for the fabrication of functionalized carbon materials. Biomasses have been considered as carbonaceous precursors because of their easier availability, higher abundance, and lower cost than those of the other carbon precursors. It can be easily transformed into carbonaceous material, which is a prospectively efficient recycling approach [4]. Another efficient way to treat coffee sludge waste is to convert it to value-added products. However, as a carbonaceous material, the waste coffee slurry is not a good precursor for conductive carbon and exhibits a low performance as an energy storage material due to its polymeric structure.

To solve this problem, we recycled the coffee slurry into hydrated carbon via sequential hydrothermal carbonization (HTC) as an activation process. The HTC reaction converts the cellulose-based biomass into hydrochar, bio-liquid, and gas at a relatively low temperature range (180 °C~230 °C) [5]. Hydrochar has various surface functional groups; therefore, it has been investigated for alternative fuels, carbon dioxide sequestration, and heavy metal immobilization for environmental remediation [6,7,8,9]. During sequential hydrothermal activation, a porous and graphitic structure of the hydrochar can be developed, where these features are the most important characteristics of materials for electron accumulation. The porous and graphitic nature of carbonaceous materials facilitates electron accumulation for capacitive reactions [10]. The electrical double-layer capacitive (EDLC) reaction has attracted considerable interest for next-generation energy storage systems such as supercapacitors. In most cases, supercapacitors combine the advantages of conventional capacitors and rechargeable batteries; they deliver high power in a short time, while providing a high specific energy [11]. Although a high degree of capacitance has been attained with certain specific structures such as ordered mesoporous carbon (OMC), graphene, or carbon nanotubes (CNTs), these materials are relatively exclusive because of the elaborate synthesis methods and inadequate precursor materials which significantly restrict the opportunity for large-scale uses [10,11,12,13]. In this context, it is highly essential to produce low-cost carbonaceous material using precursors originated from biomass [14].

Herein, we report an electrochemical investigation of coffee sludge-derived carbon electrodes as supercapacitor electrode materials. Coffee sludge was selected for investigation because it is abundant and its use alleviates environmental issues; furthermore, its intrinsic fibrous structure is expected to be beneficial for achieving suitable ionic and electronic conductivities after an activation process is completed. Among the carbon precursors, hard carbons are fundamentally not graphitizable with the amorphous nature [15], while soft carbons can be graphitized by simple heat treatment. The cellulose-based coffee sludge has a combination of both these structures, which allows partial graphitization with a random porous structure; this combination is optimal for materials targeted toward supercapacitor electrodes. The unique graphitic porous carbon derived from the waste coffee sludge is attractive due to the following reasons: it effectively resolves waste handling issues, offers a cheaper recycling method, and diminishes the requirement for landfills.

## 2. Materials and Methods

### 2.1. Chemicals and Reagents

Potassium hydroxide was purchased from Ducksan Reagent (South Korea). Distilled water and pure water were used for the experiment (18.2 MΩ•cm, PURE ROUP 50, Purewater, Korea). A filter paper with a pore size of 0.8 μm (Hyundai Micro., Seoul, Korea) and a syringe filter with a pore size of 0.2 μm (polytetrafluoroethylene (PTFE) with a glass fiber pre-filter, 13 mm ID, Echromscience, Korea) were used for filtration.

### 2.2. Generation of Hydrochar from Coffee Sludge

Waste coffee sludge (CS, T Café, Korea) was homogenized using a colander (2 mm × 2 mm). To check the moisture content, we dried the coffee sludge at 105 °C for 24 h to measure the difference in mass. Distilled water was added to the coffee sludge to adjust the moisture content to approximately 80%, which was suitable for HTC. The feedstock (coffee sludge with a moisture content of 80%; 80 g) was transferred to a hydrothermal batch reactor and the reaction was allowed to proceed for 4 h at 230 °C. The hydrochar (HC) generated after the reaction was separated by filtration. The separated hydrochar was washed with acetone and dried in an oven at 105 °C for 24 h. To activate the synthesized material, the dried hydrochar was mixed with 1 M KOH at a mass ratio of 1:3.5. The mixed sample was also heated at 700 °C for 2 h in the hydrothermal reactor. After the reaction, the activated hydrochar (AHC) was washed with pure water until a pH value of 7 was reached; subsequently, it was dried in an oven at 105 °C for 24 h. 

### 2.3. Structural Characterization

The crystal structure of CS, HC, and AHC was evaluated by Fourier-transform infrared spectroscopy (FT-IR), X-ray diffraction (XRD), nitrogen (N_2_) adsorption/desorption isotherm, scanning electron microscopy (SEM), and transmission electron microscopy (TEM). X-ray diffraction (XRD, SmartLab, Rigaku) is conducted with Cu-Kα radiation (wavelength = 1.5406 Å) at 40 kV and 30 mA to verify the amorphous structure. The morphology and surface area of as synthesized materials were determined by the Brunauer–Emmet–Teller (BET) method. The average pore sizes and pore volumes were predicted by applying the Barrett–Joyner–Halenda (BJH) formula.

### 2.4. Electrochemical Analysis

A standard three-electrode system was used. Each active material (CS, HC, AHC) was combined with carbon additive (ketjen black) as the conductor and polytetrafluoroethylene (PTFE, Nanobest) as the binder (mass ratio, 8:1:1). The mixture was coated on the surface of a stainless steel mesh as a current collector (1 cm × 1 cm). NaCl-saturated Ag/AgCl was used as a reference electrode, Pt mesh was used as a counter electrode, and 1 M Li_2_SO_4_ solution was used as an aqueous electrolyte. Cyclic voltammetry (CV), galvanostatic charge–discharge (GCD) measurement, and electrochemical impedance analysis (EIS) were conducted using an electrochemical workstation (WonA Tech, ZIVE SP1) at room temperature. CV was performed from 0 to 0.8 V while varying the scan rate in the range of 1 to 10 mVs^−1^. The GCD test was conducted on various current density from 0.05 to 0.3 A g^−1^ within the potential window of 0 to 0.8 V. The EIS frequency range was from 1 MHz to 10 µHz at an applied potential of 0.4 V and the amplitude of voltage perturbation was 10 mV.

## 3. Results and Discussion

**Figure 1:**
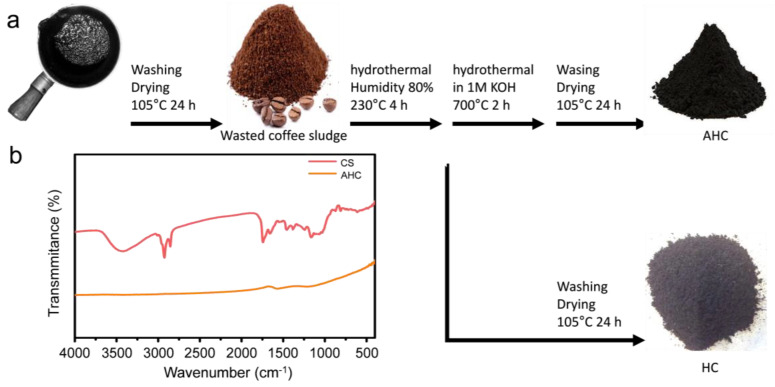
(**a**) Scheme of synthesis for hydrochar (HC) and activated hydrochar (AHC) from spent coffee sludge and (**b**) FT-IR spectrum of spent coffee sludge (CS) and AHC.

Figure 1a illustrates the scheme of the synthesis for the graphitic porous carbon from waste coffee sludge (CS), consisting of scaled hydrochar (HC). The yield of HC from the HTC reaction was 21.0% (±2.2%) and that of activated HC (AHC) was 16.0% (±10.5%). A bio-liquid was produced along with a gas with yields of approximately 63.1% (±2.9) and 15.8% (±3.6%), respectively. FT-IR was used to compare the functional groups of the feed coffee sludge and the AHC obtained after the hydrothermal reaction (Figure 1b). The absorption bands from 3600 cm^−1^ to 3100 cm^−1^ were assigned to OH stretching (from alcohols, phenols, or carboxylic acid). The peaks around 2925 cm^−1^ were assigned to the C-H asymmetric and symmetric stretch of the methylene groups and methyl groups, while the peak at 2854 cm^−1^ corresponds to the -CH stretch of the cellulose backbone [16]. The peak at 1705 cm^−1^ corresponds to the C=O vibration (ketone, aldehyde). The peaks between 1300 cm^−1^ and 1700 cm^−1^ indicate aromatic carbons. Those around 1166 cm^−1^ and 1037 cm^−1^ are attributed to C-O stretching. After the hydrothermal carbonization reaction, most of the bands were weakened, which was indicative of carbonization.

**Figure 2:**
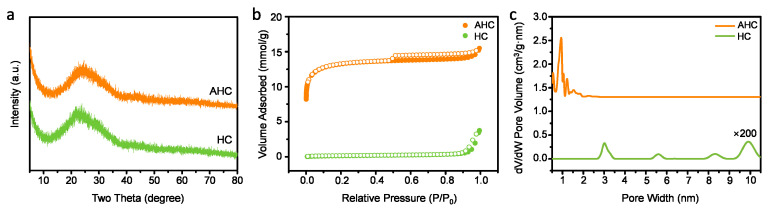
(**a**) XRD spectra of activated (AHC) and non-activated (HC) hydrochar. (**b**) N_2_ adsorption-desorption isotherms and (**c**) pore size distributions for AHC and HC.

Formation of the carbonized structure during the hydrothermal process was also proved by X-ray diffraction (XRD) (Figure 2a). In general, the XRD patterns indicate the amorphous nature of HC and AHC. However, a large broad peak at 2θ ≈ 25° was observed for both HC and AHC, which corresponds to the loose interlayer spacing of pristine graphite (3.4 Å, (002) plane) [10]. This characteristic peak originates from partial graphitization of the glucose ring in the cellulose backbone of waste coffee sludge. Graphitization is helpful for an effective charge transfer in supercapacitors because of the inherent conducting feature of graphitic carbon. 

To gain deeper insights into the microstructure of HC and AHC with regard to the specific surface area and pore size distribution, N_2_ adsorption/desorption isotherms were conducted (Figure 2b,c). As shown in Figure 2b, the N_2_ sorption isotherm of AHC is a typical IV isotherm that is based on the IUPAC classification, suggesting a highly developed pore structure in the AHC composite [17]. The results show that the KOH activation process reduces the regularity of the pre-cellulose structure due to the formation of pores or defects [18], and the pore structure of AHC confers a higher surface area as compared to that of the non-activated HC [19,20]. The relatively fewer number of macropores may also be advantageous because they form a part of the carrying zone in the interconnection of the porous structure [4]. To evaluate the pore structure, the pore size distribution was studied using the BJH method (Figure 2c). 

As shown in Figure 2c, AHC has a pore diameter of less than 2 nm, which is indicative of a microporous structure. The dimensional similarity of the pores and ions in the electrolyte has a critical role for the EDLC mechanism. The dimensions of the Li^+^ and SO_4_^2−^ components of the electrolyte used herein are in the range of 0.38 nm, which can improve the energy efficiency of EDLC mechanism when AHC is used as electrodes [21,22,23]. The total specific surface area was considered from Brunauer–Emmett–Teller (BET) equation. As expected, the specific surface area of the AHC sample (1067.114 m^2^ g^−1^) was significantly higher than that of HC (7.0164 m^2^ g^−1^). 

**Figure 3:**
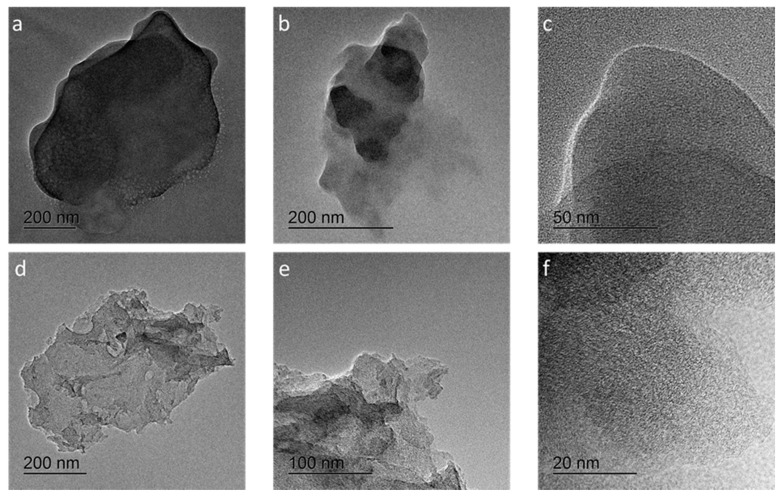
TEM images of (**a**) coffee sludge (CS), (**b,c**) non-activated hydrochar (HC), and (**d**–**f**) activated hydrochar (AHC). High-resolution TEM images of individual AHC showing torn porous structure and rough graphitic structure.

**Figure 4:**
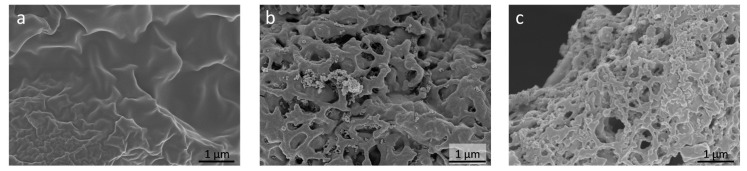
SEM images of (**a**) coffee sludge (CS), (**b**) non-activated hydrochar (HC), and (**c**) activated hydrochar (AHC).

The TEM images are in good agreement with the observed BET data. The TEM images in Figure 3 show that a porous and graphitic structure was obtained after the activation process. The (002) lattice fringes of the rough graphitic structure were clearly observed in the HR-TEM images (Figure 3f). Figure 4 shows the SEM images of raw coffee sludge waste, HC, and AHC. The surface of the raw coffee sludge is slightly wrinkled. After the hydrothermal carbonization reaction, the surface becomes rougher with macropores due to the decomposition of the components. Notably, both HC and AHC had a macroporous surface structure; however, the sequential activation process generates hierarchical micropores on the macrostructured surface that leads to the formation of a unique hierarchical porous structure, thereby maximizing the electroactive surface area.

**Figure 5:**
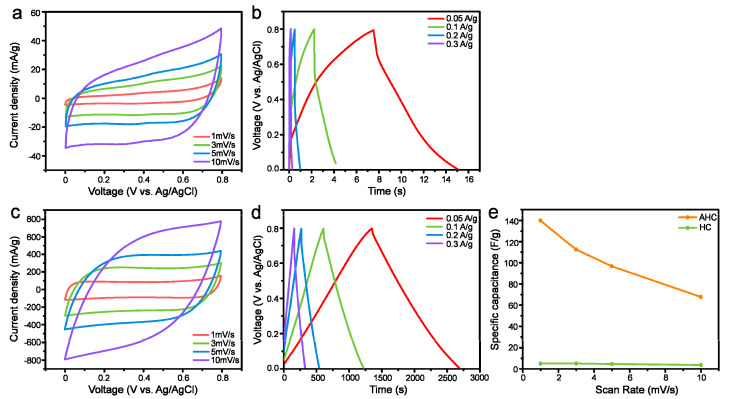
(**a**) CVs of HC electrode from 1 to 10 mV s^−1^. (**b**) Charge–discharge curves of HC electrode from 0.05 to 0.3 A g^−1^. (**c**) CVs of AHC electrode from 1 to 10 mV s^−1^. (**d**) CD graphs of AHC electrode from 0.05 A g^−1^ to 0.3 A g^−1^. (**e**) Specific capacitance of AHC and HC from the galvanostatic discharge curves at different scan rates.

In this context, AHC is a remarkable material for manufacturing EDLC electrodes. The micropores have a high surface-to-volume ratio, which enhance the adsorption–desorption of electrolyte species through diffusion on electrode and provide good adsorbate approachability via the wide carrying networks [24]. Electrochemical analyses of the prepared materials were conducted to evaluate their performance as EDLC electrodes; the results have been shown in Figure 5. CVs of the HC and AHC electrodes were acquired at scan rates ranging from 1 to 10 mV s^−1^. As shown in Figure 5a,c, the CVs of HC and AHC display a quasi-rectangular shape from 1 to 5 mV s^−1^, wherein the current increases almost linearly with the scan rate, indicating an excellent EDLC behavior [8]. Although a resistive behavior was observed at higher scan rates, the CVs also retained a reasonable shape. On the other hand, without the activation process, the specific capacitance decreased significantly. This observation suggests that the porosity and conductivity were significantly improved by the activation of HC; this result was in good agreement with the morphological characteristics. It is highly probable that AHC acts as a tunnel for ion and electron transfer, as assumed and shown in Figure 3 and Figure 4. Figure 5b,d show the GCD profiles of the HC and AHC electrode at various current densities from 0.05 to 0.3 A g^−1^. The GCD curves of AHC had a symmetrical triangular shape with small internal resistance (IR drop), indicating a good coulombic efficiency in the charge and discharge. The slight deviation of the charge and discharge curves from linearity indicates a difference in the ion and electron conductivities of HC and AHC. The equivalent series resistance (ESR) can be calculated from the IR drop (*V_drop_*) with a constant current density (*I_cons_*) based on Equation (1).

(1) $$R_{ESR}=\frac{V_{drop}}{2\times I_{cons}}$$

The equivalent series resistance (ESR) of HC and AHC estimated from the IR drop was 134 and 33 mΩ at 0.05 A g^−1^, respectively. The IR drop was lower for AHC than that observed for HC, which may be due to a decrease in the internal resistance of the electrode materials [25]. The observation of an IR drop at the initial point of the discharge curve shows the resistance of the electrolytes and the inner resistance of the electrode materials. The latter usually has a greater contribution to the overall IR drop [26]. The relationship between specific capacitance and scan rate was shown in Figure 5e, indicating a decrease of the capacitance for HC is relatively severe compared to that of AHC with increasing scan rate. Such behavior can be attributed to two reasons, namely a reduced penetration of the ions into the pores and the increased ion diffusion resistance in electrolytes at a high scan rate [27]. As shown in Figure 5b,d, the supercapacitive performance of AHC is greater than that of HC. Based on the comparison in Figure 5b,d, the discharging time of AHC is definitely longer than that of HC, which indicates that the former exhibits a higher specific capacitance. The specific capacitances of HC and AHC have been compared in Figure 5e. The specific capacitance of AHC was estimated from the GCD curves and the values of 140, 113, 97, and 68 F g^−1^ were obtained at the scan rates of 1, 3, 5, and 10 mV s^−1^, respectively; these values were higher than those of the HC electrodes. It should be noted that the specific capacitance of AHC at 10 mV s^−1^ (68 F g^−1^) exceeded the value for HC at 1 mV s^−1^ scan rate (5 F g^−1^). 

**Figure 6:**
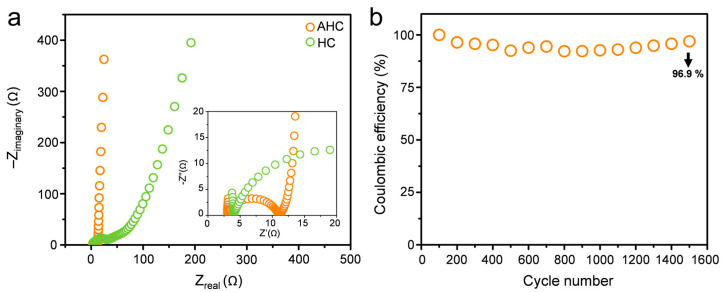
(**a**) Nyquist plots of AHC and HC at applied potential of −0.4 V for AC amplitude of 10 mV in the range from 1 MHz to 10 µHz. (**b**) Cycle life of AHC electrode over 1500 cycles at current density of 0.3 A g^−^^1.^

The enhanced electrochemical conductivity of the AHC materials have been further investigated via EIS measurements; the corresponding Nyquist plots for AHC and HC are presented in Figure 6. The observed spectra exhibit different behavior in various frequency regions. The high frequency region of the EIS spectra is expanded in the inset of Figure 6b. The diameter of the semicircle implies charge transfer resistance (*R_ct_*), which corresponds to the sum of the contact resistance between the electrode and the conductor, electrolyte resistance in the porous electrode structure, and electrode resistance [28,29,30]. The charge transfer resistance of HC (32.6 Ω) is higher than that of AHC (11.0 Ω). A smaller semicircle implies that the charge transfer is faster and that the resistance is smaller. This means that activation of the carbon improved the charge transfer at the electrode/conductor surface interface. Owing to the continuous connection of the AHC particles therein, the conductivity of AHC is superior to that of HC; this is expected because AHC has a connected graphitized structure, which is not observed for HC, as confirmed by the TEM data (Figure 3). The tangent to the curve in the low frequency region for AHC is higher than that for HC. This suggests a better ion propagation and more ideal supercapacitive performance of the former. Figure 3 and Figure 4 show the morphological differences due to the activation reaction, indicating the existence of a well-developed hierarchical porous structure in AHC as compared to that the structure of HC. When the porous electrode is dipped into the electrolyte, the interconnections between the macropores not only provide sites for localization of the electrolyte, but also form pathways for ion diffusion. The cycling stability is another key parameter that determines the practical application of supercapacitors. The cycle stability of the AHC electrode was tested in 1 M Li_2_SO_4_ electrolyte at a current density of 0.3 A g^−1^. As shown in Figure 6b, the specific capacitance of the AHC electrode was sustained at 51 F g^−1^ after 1500 successive cycles. This indicates that the specific capacitance was reduced by only 3% after 1500 cycles, which shows that the AHC electrode is suitable for practical applications.

## 4. Conclusions

Graphitic porous carbon was synthesized via a simple sequential hydrothermal method using the waste coffee sludge. The graphitic porous carbon, which is derived from spent coffee sludge, was employed in EDLCs by manipulating its unique microstructure which has a high hierarchical porous nature and graphitic edges. Because of the high surface area (~1067 m^2^ g^−1^) and a hierarchical multi-porous structure, AHC gives easy pathways for ion and electron transport, which provides a higher electrochemical performance as an EDLC electrode than that demonstrated by the HC. The specific capacitance of the AHC-based supercapacitors was 140 F g^−1^ and the supercapacitors demonstrated a good cyclic performance over 1500 cycles at current density of 0.3 A g^−^^1^. Thus, a very efficient and low-cost method for preparing AHC from waste coffee sludge was developed, which could be an environmentally friendly technique for the recycling of approximately eight million metric tons of cellulose-based waste and for applications associated with energy storage systems.
